# Dietary Curcumin Supplementation Increases Antioxidant Capacity, Upregulates Nrf2 and Hmox1 Levels in the Liver of Piglet Model with Intrauterine Growth Retardation

**DOI:** 10.3390/nu11122978

**Published:** 2019-12-05

**Authors:** Yu Niu, Jintian He, Hussain Ahmad, Mingming Shen, Yongwei Zhao, Zhending Gan, Lili Zhang, Xiang Zhong, Chao Wang, Tian Wang

**Affiliations:** 1 College of Animal Science and Technology, Nanjing Agricultural University, Nanjing 210095, China; niuyu0227@126.com (Y.N.);; 2 Animal Nutrition Department, University College of Veterinary and Animal Sciences, The Islamia University Bahawalpur, Bahawalpur 63100, Pakistan

**Keywords:** curcumin, intrauterine growth retardation, piglets, antioxidant capacity, liver

## Abstract

Curcumin has improved effects on antioxidant capacity via multiple mechanisms. Intrauterine growth retardation (IUGR) has had adverse influences on human health. IUGR is always associated with elevated oxidative stress and deficiencies in antioxidant defense. Therefore, we chose IUGR piglets as a model to investigate the effects of IUGR on antioxidant capacity of newborn and weaned piglets and determine how these alterations were regulated after supplementation with curcumin in weaned IUGR piglets. In experiment 1, eight normal-birth-weight (NBW) and eight IUGR newborn piglets were selected to determine the effect of IUGR on the antioxidant capacity of neonatal piglets. In experiment 2, thirty-two weaned piglets from four experimental groups: NBW, NC (curcumin supplementation), IUGR, IC (curcumin supplementation) were selected. The results showed that both IUGR newborn and weaned piglets exhibited oxidative damage and lower antioxidant enzymes activities in the liver compared with the NBW piglets. Dietary curcumin supplementation increased body-weight gain, feed intake, activities of antioxidant enzymes, and the expressions of nuclear factor, erythroid 2-like 2 (Nrf2) and heme oxygenase-1 (Hmox1) proteins in the liver of weaned piglets with IUGR. In conclusion, IUGR decreased the antioxidant capacity of newborn and weaned piglets. Curcumin could efficiently improve the growth, increase hepatic antioxidant capacity, and upregulate Nrf2 and Hmox1 levels in the liver of IUGR weaned piglets.

## 1. Introduction

Curcumin (C_21_H_20_O_6_), a hydrophobic polyphenol extracted from the rhizome of the herb *Curcuma longa*, has a wide range of biological and pharmacological activities [1]. In human clinical trials, it is suggested that the curcumin is effective and can be used in a dosage of 8 g/d [2]. It has been proved that the curcumin revealed many medicinal properties such as antioxidant, antiapoptotic, proliferative properties [3,4], anti-inflammatory [5], anticancer, antitumor activities, and confirmed the protective effects against various diseases [6]. Previous study found that dietary curcumin supplementation had beneficial effects on improving the growth performance of weaned pigs [7]. The positive role of curcumin in modulating hippocampal nitric oxide production has been well reported in pig research [8]. In recent years, Gao et al. [9] revealed that curcumin can also increase antioxidant capacity in mice with oxidative injury through activation of the Nrf2 pathway.

Intrauterine growth retardation (IUGR) is usually defined as a failure of normal growth and development of fetus and/or its organs during gestation [10]. The IUGR neonates have lower birth weight than normal neonates, mainly due to malnutrition caused by placental insufficiency. IUGR has become a serious problem in animal production. Furthermore, it has numerous adverse impacts on the growth and development of animals after birth [11,12]. It negatively influences neonatal survival, postnatal growth performance, feed utilization, normal functioning of tissues or organs, and is detrimental to health in the long term to the body in adults. Amarilyo et al. [13] observed that infants with IUGR had more risk of inflammation. Previous studies have shown that pregnant women with IUGR fetuses had higher oxidative stress and poorer antioxidant defense system [14]. It has been demonstrated that levels of lipid peroxidation and protein oxidation in the erythrocyte were increased, both in IUGR fetuses and their mothers [15]. These studies indicated that the poor antioxidant defense found in in utero stunted newborns. Previous studies revealed that IUGR impaired hepatic antioxidant capacity [16] and resulted in oxidative damage [17] in weaned piglets.

However, studies that describe the antioxidant capacity of IUGR neonatal piglets and treatment of curcumin on weaned IUGR piglets are currently very limited. In the present study, we have chosen the piglet model because it is widely accepted that pigs possess a gastrointestinal system very similar to that of humans. The present study is, to the best of our knowledge, the first attempt to elucidate the effects of curcumin on the antioxidant capacity of IUGR piglets. Therefore, the aim of this study was to investigate whether IUGR could decrease antioxidant capacity in the liver of neonatal piglets. Based on the consideration of animal welfare, suckling piglets should be nursed by their own maternal sows. We thought that curcumin is more suitable as a dietary supplement for weaned piglets. Then, we chose weaned piglets with IUGR to determine the influences of dietary supplementation of 400 mg curcumin/kg on the hepatic antioxidant activity of IUGR weaned piglets in order to provide new nutrient strategies for IUGR.

## 2. Materials and Methods

### 2.1. Curcumin Preparation

The curcumin used in this study was kindly provided by Kehu Bio-technology Research Center (Guangzhou, China; website: http://www.co-hoo.com). The content of curcumin was 98% as determined by HPLC analysis.

### 2.2. Animal Experiment Design

The experimental design and procedures were approved by the Institutional Animal Care and Use Committee of Nanjing Agricultural University following the requirements of the Regulations for the Administration of Affairs Concerning Experimental Animals of China (NJAU-CAST-2018-023). The animal trials were conducted on experimental pig farm, owned by the Jiangsu Lihua Animal Husbandry Co., Ltd. (Suqian, Jiangsu Province, China). The sows, with similar birth order (third or fourth), fed with the same gestating diet that met National Research Council (NRC, 2012) nutrient requirements [18]. On the day of delivery, the birth weight and sex of each newborn piglet were recorded.

In experiment 1, eight normal-birth-weight (NBW) and eight IUGR newborn piglets (Duroc × (Landrace × Yorkshire)) were selected from eight litters, one NBW and one IUGR newborn piglet per litter. In each litter, piglets with birth weights of 1.52 ± 0.03 kg (mean ± standard deviation (SD); within one SD of the mean birth weight) and 0.81 ± 0.03 kg (mean ± SD; two SD below the mean birth weight) were selected as NBW and IUGR piglets according to previous studies [19,20], respectively. A total of 16 neonatal piglets (half male and half female) were stunned by electric shock and killed by jugular bloodletting within 1 hour after birth without suckling.

In experiment 2, the selection of NBW and IUGR newborn piglets was similar to that in experiment 1. A total of 20 NBW and 20 IUGR newborn piglets were obtained from 20 litters, one NBW and one IUGR newborn piglets per litter. In each litter, piglets with birth weights of 1.51 ± 0.04 kg (mean ± SD; within one SD of the mean birth weight) and 0.96 ± 0.02 kg (mean ± SD; two SD below the mean birth weight) were defined as NBW and IUGR piglets and marked by different tags, respectively. Piglets were limited to 11/litter to normalize rearing (average 12.1 piglets/litter). During the 26-day lactation period, sows weaned their own piglets. On day 26, all piglets were weighed and allocated to four groups. In each group, piglets were assigned in five boxes (two animals in each box, one male and one female). The NBW piglets were randomly assigned to the NBW and NC (curcumin supplementation) groups (*n* = 10/group, five males and five females), and IUGR piglets were randomly assigned to the IUGR and IC (curcumin supplementation) groups (*n* = 10/group, five males and five females). The NBW and IUGR groups were fed with basal diets, and the NC and IC groups were fed with basal diets supplemented with 400 mg curcumin/kg until day 50. The diets supplemented with 400 mg curcumin/kg was according to the previous study [7]. They demonstrated that dietary supplementation of 400 mg curcumin/kg was more effective in improving the health status of weaned pigs. All piglets were housed individually at an ambient temperature of 25–28 °C and had free access to water. At 50 d of age, piglets were weighed after feed deprivation for 12 h to calculate total body-weight gain (BWG), and feed consumption was recorded daily by box to calculate total feed intake (FI) and feed conversion ratio (G:F, BWG:FI). The compositions of the diets are presented in Table S1. A total of 32 piglets with nearly similar body weight within group (eight piglets/group, half male and half female) were stunned by electric shock and killed by jugular bloodletting at the end of the experiment.

### 2.3. Sample Collection

At 0 d of age in experiment 1 and 50 d of age in experiment 2, blood samples were obtained by jugular venipuncture and then centrifuged at 3000× *g* for 15 min at 4 °C. The serum was stored at −20 °C to keep the contents stable and for further analyses. The piglets were killed in the order of one piglet per group to avoid the effect of time. In both experiments, fresh liver tissue samples (the same right lobe area) were immediately collected using ice cubes and then stored at −80 °C in order to avoid the degradation of RNA and proteins and for further analyses.

### 2.4. Analysis of Serum Parameters

Serum lipid peroxidation level was expressed by malondialdehyde concentration (MDA Concentration Testing Kit, no. A003), which is a byproduct of lipid peroxidation. Concentrations of MDA and hydrogen peroxide (H_2_O_2_ Concentration Testing Kit, no. A064-1), activities of total antioxidant capacity (TAOC Activity Testing Kit, no. A015-1), catalase (CAT Activity Testing Kit, no. A007-1), glutathione peroxidase (GSH-Px Activity Testing Kit, no. A005), and glutathione reductase (GR Activity Testing Kit, no. A062) in the serum were determined according to the manufacturer’s instructions of Nanjing Jiancheng Bioengineering Institute (Nanjing, Jiangsu Province, China). The detailed instructions of these testing kits are clearly described in our supplemental files (https://doi.org/10.5281/zenodo.3520037). Serum activities of aspartate aminotransferase (AST) and alanine aminotransferase (ALT) were determined according to the previous study (Selecta XL; Vital scientific, Newton, MA, USA) [21].

### 2.5. Analysis of Liver Antioxidant Status

The frozen liver samples (0.4 g) from −80 °C were homogenized with a handheld homogenizer (Pro 200; Pro Scientific Inc., Oxford, CT, USA) in 0.86% (w/v) ice-cold physiological saline (3.6 mL) or tissue homogenate provided by the corresponding diagnostic kit (Nanjing Jiancheng Bioengineering Institute, Jiangsu, China) according to the instructions of the manufacturer. The homogenate was centrifuged at 3500× *g* for 15 min at 4 °C and the supernatants were immediately collected and stored at −20 °C for measurement. Protein contents of liver were measured using the bicinchoninic acid (BCA) protein assay of Nanjing Jiancheng Bioengineering Institute (Nanjing, Jiangsu Province, China; BCA Assay Kits, no. A045-3). Protein oxidation in the liver was measured via the concentration of protein carbonyl (PC Concentration Testing Kit, no. A087). Concentrations of MDA (MDA Concentration Testing Kit, no. A003), H_2_O_2_ (H_2_O_2_ Concentration Testing Kit, no. A064-1), glutathione (GSH Concentration Testing Kit, no. A006), oxidized glutathione (GSSG Concentration Testing Kit, no. A061-2), glutathione reductase (GR Concentration Testing Kit, no. A062), and activities of CAT (CAT Activity Testing Kit, no. A007-1), TAOC (TAOC Activity Testing Kit, no. A015-1), GSH-Px (GSH-Px Activity Testing Kit, no. A005), total nitric oxide synthase (TNOS Activity Testing Kit, no. A014-2), and inducible nitric oxide synthase (iNOS Activity Testing Kit, no. A014-1) in the liver were measured according to the manufacturer’s instructions of Nanjing Jiancheng Bioengineering Institute (Nanjing, Jiangsu Province, China). The detailed instructions for these testing kits are clearly described in our supplemental files (https://doi.org/10.5281/zenodo.3520037).

### 2.6. Gene Expression

Total RNA from the liver samples at 0 and 50 days of age stored at −80 °C was isolated using the Trizol reagent (Invitrogen, Shanghai, China). The determination of RNA content, mRNA quantification and real-time PCR (Applied Biosystems) were performed according to previously described methods [22]. Briefly, RNA was quantified based on the absorption of light at 260 nm (A260) and 280 nm (A280). The RNA quality was assessed by agarose gel electrophoresis. The primer sequences for the target and housekeeping genes used for real-time PCR are listed in Table S2. *Gapdh* was also used as a control gene to normalize the expression of target genes (similar results were obtained and only showed the results using *β-actin* as a normalizer). Briefly, a reaction system of 20 μL was composed of 0.4 μL of forward primers, 0.4 μL of reverse primers, 0.4 μL of ROX Reference Dye, 10 μL of SYBR Premix Ex Taq (TaKaRa Biotechnology Co. Ltd., Dalian, China), 6.8 μL of double-distilled water, and 2 μL of complementary DNA. The 2^−ΔΔCt^ method was used to calculate relative levels of mRNA expression after normalization with housekeeping genes [23]. The values for the NBW group were used for calibration.

### 2.7. Western Blotting

Antibodies against total nuclear factor, erythroid 2-like 2 (Nrf2, dilution 1:500; catalog no. 16396-1-AP; Source: rabbit) and Hmox1 (dilution 1:500; catalog no. 27282-1-AP; source: rabbit) were purchased from Proteintech Group (Rosemont, IL, USA). Antibodies against *β*-actin (dilution 1:2000; catalog no. 20536-1-AP; source: rabbit) were purchased from Proteintech Group (Rosemont, IL, USA). The proteins of the liver were extracted using assay kits according to the manufacturer’s instructions (Beyotime Institute of Biotechnology, Shanghai, China; no. P0013B). The protein content of each sample was assayed using the BCA Protein Assay Kit (Nanjing Jiancheng Bioengineering Institute, Jiangsu, China; no. A045-3). For western blotting analyses, 40 µg of protein from each sample was subjected to sodium dodecylsulfate-polyacrylamide gel electrophoresis. After electrophoresis, proteins were separated and transferred to polyvinylidene difluoride membranes. The membranes were blocked with blocking buffer (5% non-fat dry milk) for 2 h at room temperature. The membranes were then probed with appropriate primary and secondary antibodies (horseradish peroxidase-conjugated goat anti-rabbit immunoglobulin G, Proteintech Group; 1:5000 dilution in 1 × TBS with 0.1% Tween 20; catalog no. SA00001-2). The blots were detected using enhanced chemiluminescence reagents (ECL-Kit, Beyotime Institute of Biotechnology, Jiangsu Province, China) followed by autoradiography. Photographs of the membranes were taken using the Luminescent Image Analyzer LAS-4000 system (Fujifilm Co. Ltd., Tokyo, Japan) and quantified by Gel-Pro Analyzer 4.0 software (Media Cybernetics, Bethesda, MD, USA).

### 2.8. Statistical Analysis

The data all accorded with normality by using Kolmogorov–Smirnov (K-S) test (SPSS 17.0; SPSS, Inc., Chicago, IL, USA) [24]. In experiment 1, data were analyzed using unpaired, independent *t*-tests. In experiment 2, data were analyzed using a two-way analysis of variance. The classification variables were birth weight (NBW + NC × IUGR + IC), diet (NBW + IUGR × NC + IC), and the interaction between birth weight and diet (NBW × NC × IUGR × IC). A Tukey’s post hoc analysis was used to determine the differences between the four groups when a statistically significant birth weight × diet interaction was observed. SPSS 17.0 (SPSS, Inc., Chicago, IL, USA) was used for these analyses. A probability level of *p* < 0.05 was considered statistically significant, and *p* < 0.01 was considered very significant. Data were presented as mean ± standard deviation.

## 3. Results

### 3.1. Growth Performance

In experiment 1, the birth weight of IUGR piglets was significantly lower (*p* < 0.01) than that of NBW piglets (0.81 ± 0.03 kg vs. 1.52 ± 0.03).

In experiment 2, IUGR piglets showed lower (*p* < 0.01) body-weight gain (BWG) and feed intake (FI) compared to NBW piglets (Table 1). The BWG and FI of IC group were higher (*p* < 0.05) than those of IUGR group.

### 3.2. Serum Antioxidant Capacity of Newborn and Weaned Piglets

**Figure 1:**
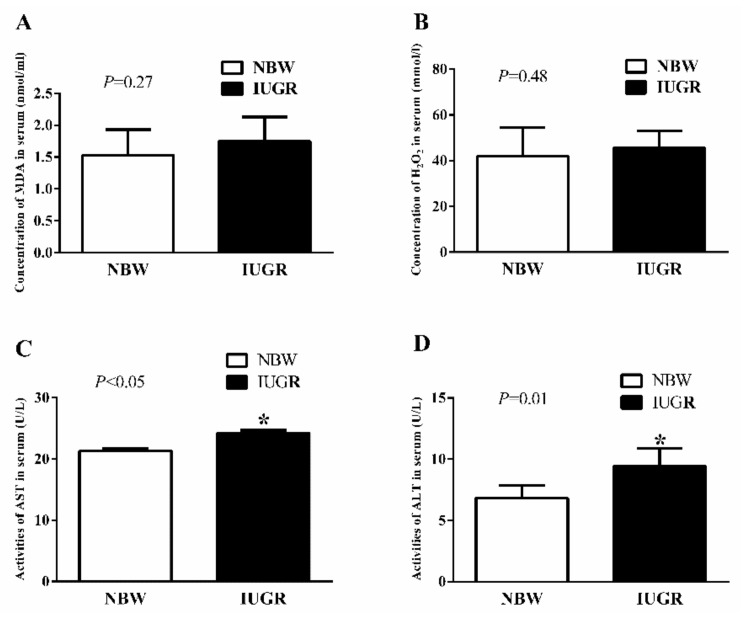
Effects of intrauterine growth retardation on the concentrations of MDA (**A**) and H_2_O_2_ (**B**), and activities of AST (**C**) and ALT (**D**) in the serum of newborn piglets (0 d). Values expressed as mean ± standard deviation, *n* = 8/group. Data were analyzed using unpaired independent *t*-tests. * A significant difference was observed (*p* < 0.05). NBW, normal birth weight; IUGR, intrauterine growth retardation; MDA, malondialdehyde; H_2_O_2_, hydrogen peroxide; AST, aspartate aminotransferase; ALT, alanine aminotransferase.

In experiment 1, IUGR newborn piglets had no significant differences of serum malondialdehyde (MDA, *p* = 0.27) and hydrogen peroxide (H_2_O_2_, *p* = 0.48) compared with the NBW piglets. The activities of serum aspartate aminotransferase (AST, *p* < 0.05) and alanine aminotransferase (ALT, *p* = 0.01) in the IUGR newborn piglets were significantly higher than those in the NBW piglets (Figure 1).

**Figure 2:**
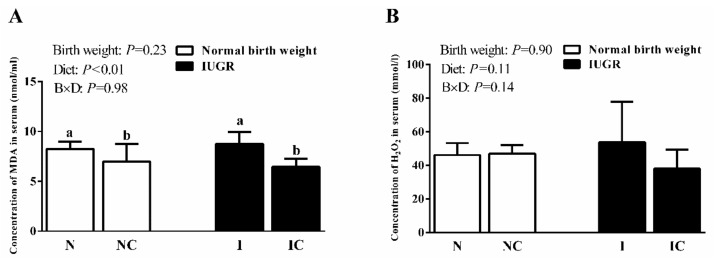
Concentrations of MDA (**A**) and H_2_O_2_ (**B**) in the serum of normal-birth-weight piglets (N), normal-birth-weight piglets supplemented with curcumin (NC), intrauterine growth retardation piglets (I), intrauterine growth retardation piglets supplemented with curcumin (IC) (50 d). Values expressed as mean ± standard deviation, *n* = 8/group. Data were analyzed by using two-way analysis of variance. Significant differences marked with different letters when a significant interaction was observed (*p* < 0.05). B × D was the interaction between the corresponding parameters. MDA, malondialdehyde; H_2_O_2_, hydrogen peroxide.

In experiment 2, dietary curcumin supplementation significantly decreased (*p* < 0.01) the concentration of serum MDA in the NC or IC groups compared with that in the NBW or IUGR groups (Figure 2). The activities of serum catalase (CAT), total antioxidant capacity (TAOC), and glutathione peroxidase (GSH-Px) in the IUGR group were lower (*p* < 0.05) than those in the NBW group. Because of dietary curcumin supplementation, the activities of serum CAT, TAOC, and glutathione reductase (GR) were significantly higher (*p* < 0.05) in the NC and IC groups than in the NBW and IUGR groups (Table 2).

### 3.3. Hepatic Antioxidant Capacity of Newborn and Weaned Piglets

**Figure 3:**
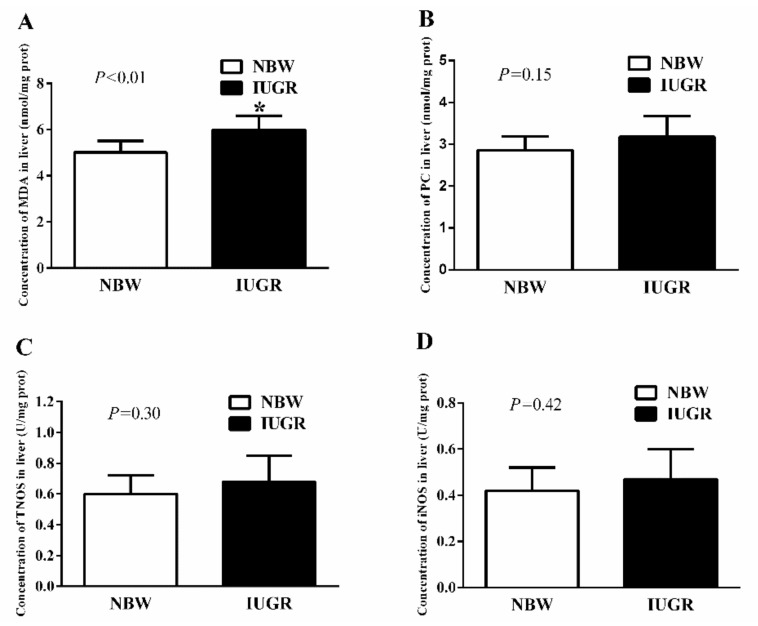
Effects of intrauterine growth retardation on the concentrations of MDA (**A**) and PC (**B**), activities of TNOS (**C**) and iNOS (**D**) in the liver of newborn piglets (0 d). Values are expressed as mean ± standard deviation, *n* = 8/group. Data were analyzed using unpaired independent *t*-tests. * a significant difference was observed (*p* < 0.05). NBW, normal birth weight; IUGR, intrauterine growth retardation; MDA, malondialdehyde; PC, protein carbonyl; TNOS, total nitric oxide synthase; iNOS, inducible nitric oxide synthase.

In experiment 1, IUGR newborn piglets had significantly higher (*p* < 0.01) MDA concentration than NBW newborn piglets. However, there were no significant differences in hepatic protein carbonyl (PC, *p* = 0.15), total nitric oxide synthase (TNOS, *p* = 0.30), and inducible nitric oxide synthase (iNOS, *p* = 0.42) levels between IUGR and NBW newborn piglets (Figure 3). The value of GSSG: GSH ratio was higher (*p* < 0.05) and activities of TAOC, GSH-Px, and GR were lower (*p* < 0.05) in the liver of IUGR newborn piglets than those in the NBW newborn piglets (Table 3).

**Figure 4:**
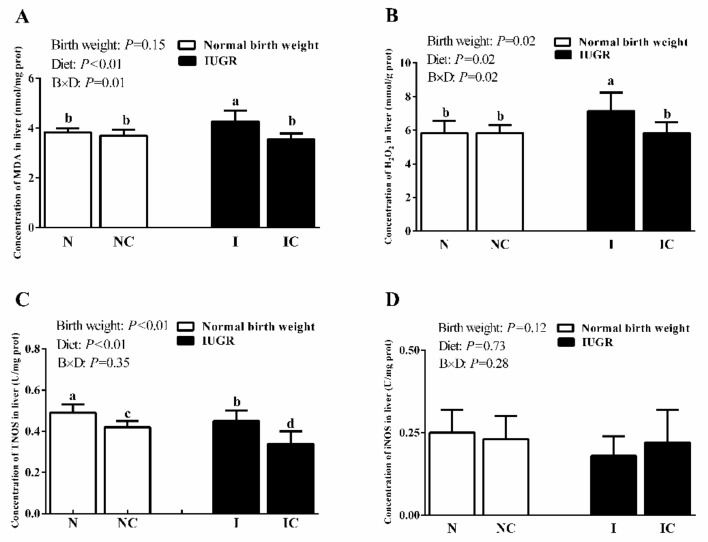
Concentrations of MDA (**A**) and H_2_O_2_ (**B**), activities of TNOS (**C**) and iNOS (**D**) in the liver of normal-birth-weight piglets (N), normal birth weight piglets supplemented with curcumin (NC), intrauterine growth retardation piglets (I), intrauterine growth retardation piglets supplemented with curcumin (IC) (50 d). Values are expressed as mean ± standard deviation, *n* = 8/group. Data were analyzed by using two-way analysis of variance. Significant differences are marked with different letters when a significant interaction was observed (*p* < 0.05). B × D was the interaction between the corresponding parameters. MDA, malondialdehyde; H_2_O_2_, hydrogen peroxide; TNOS, total nitric oxide synthase; iNOS, inducible nitric oxide synthase.

In experiment 2, IUGR weaned piglets showed significantly higher (*p* < 0.05) concentrations of MDA and H_2_O_2_, and significantly lower (*p* < 0.05) activity of TNOS in the liver compared to the NBW weaned piglets. The MDA and H_2_O_2_ concentrations and activity of TNOS were significantly decreased (*p* < 0.05) in the livers of the IC group than in the livers of the IUGR group (Figure 4). The activities of AST and ALT in the serum of IUGR weaned piglets were significantly higher (*p* < 0.05) than those of NBW weaned piglets. Diets supplemented with curcumin could significantly decrease (*p* < 0.05) the activities of AST and ALT in the serum of IUGR weaned piglets. The activity of serum AST in the NC group was significantly lower (*p* < 0.05) than that in the NBW group. The CAT activity was significantly higher (*p* < 0.05), however, GSH-Px activity was significantly lower (*p* < 0.05) in the liver of IUGR group than that of NBW group. The CAT activity was significantly lower (*p* < 0.05), while, activity of GSH-Px was significantly higher (*p* < 0.05) in the liver of IC group than in the liver of IUGR group. Furthermore, the activity of hepatic GR was significantly higher (*p* < 0.05) in the NC and IC groups than in the NBW and IUGR groups (Table 4).

### 3.4. Hepatic Nuclear Factor, Erythroid 2-Like 2 (*Nfe2l2*), Heme Oxygenase-1 (*Hmox1*), *Cat*, and Glutathione Peroxidase 1 (*Gpx1*) Gene Expressions of Newborn and Weaned Piglets

In the liver of IUGR newborn piglets, the mRNA expressions for nuclear factor, erythroid 2-like 2 (*Nfe2l2*), heme oxygenase-1 (*Hmox1*), catalase (*Cat*), and glutathione peroxidase 1 (*Gpx1*) were significantly lower (*p* < 0.01) than those of the NBW newborn piglets (Table 5). In experiment 2, as compare with NBW group, IUGR reduced (*p* < 0.05) the hepatic *Nfe2l2*, *Hmox1*, *Cat*, and *Gpx1* mRNA expressions (Table 6). Diets supplemented with curcumin enhanced (*p* < 0.05) the hepatic *Nfe2l2* and *Gpx1* mRNA expressions in the IC group. In the NC group, the hepatic *Cat* mRNA expression was enhanced (*p* < 0.05) compared to thatof the NBW weaned piglets.

### 3.5. Immunoblotting

**Figure 5:**
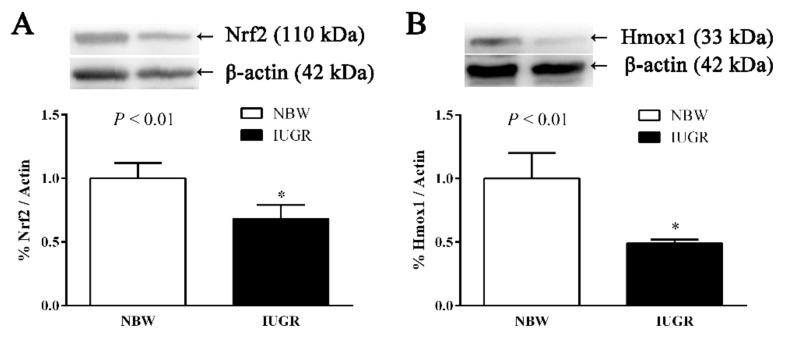
Effects of intrauterine growth retardation on the expression of Nrf2 (**A**) and Hmox1 (**B**) in the liver of newborn piglets (0 d). Values are expressed as mean ± standard deviation, *n* = 8/group. Data were analyzed using unpaired independent *t*-tests. * A significant difference was observed (*p* < 0.05). NBW, normal birth weight; IUGR, intrauterine growth retardation. Nrf2, nuclear factor, erythroid 2-like 2; Hmox1, heme oxygenase 1.

**Figure 6:**
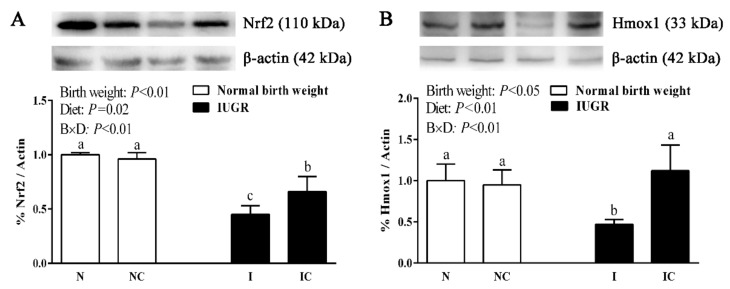
Expressions of Nrf2 (**A**) and Hmox1 (**B**) in the liver of normal-birth-weight piglets (N), normal-birth-weight piglets supplemented with curcumin (NC), intrauterine growth retardation piglets (I), intrauterine growth retardation piglets supplemented with curcumin (IC) (50 d). Values are expressed as mean ± standard deviation, *n* = 8/group. Data were analyzed by using two-way analysis of variance. Significant differences are marked with different letters when a significant interaction was observed (*p* < 0.05). B × D was the interaction between the corresponding parameters. Nrf2, nuclear factor, erythroid 2-like 2; Hmox1, heme oxygenase 1.

IUGR piglets had significantly lower (*p* < 0.05) hepatic Nrf2 and Hmox1 levels compared to the NBW piglets at 0 d (Figure 5) and 50 d (Figure 6) of age. Dietary curcumin supplementation significantly increased (*p* < 0.05) the hepatic Nrf2 and Hmox1 levels of weaned piglets with IUGR (Figure 6).

## 4. Discussion

During the last decades, many studies have described the impairment of IUGR [25] and found potential approaches to mitigate and counteract the detrimental effects of IUGR on the antioxidant defense system in animal production [16]. The pig is widely used as an animal model for human research in recent years. It has been demonstrated that oxidative and antioxidative status of IUGR were altered in weaned piglets [17]. However, there were few studies on the antioxidant capacity in newborn piglets with IUGR and designing alternative nutritional therapeutic strategies will be beneficial for the prevention and treatment of IUGR piglets.

In the present study, IUGR significantly reduced body-weight gain and feed intake of weaned piglets and dietary curcumin supplementation improved the growth performance. These findings were consistent with the previous studies that IUGR piglets showed slow growth before [20] and after weaning [26]. Similarly, diet supplemented with curcumin obviously increased the body-weight gain and feed intake of broilers under heat stress [27]. The improved effects of curcumin on the growth performance of IUGR piglets coincided with the study of Xun et al. [7], who found that dietary addition of 400 mg/kg curcumin had significant improvement on growth of weaned piglets. The present study also found that the activities of serum AST and ALT were all elevated in IUGR newborn and weaned piglets. Once the liver is damaged, AST and ALT will flow into the blood and the high levels of these two in the serum have been widely accepted as a biomarker of hepatic damage [28]. In the present study, curcumin exhibited the beneficial effect on attenuating liver injury of IUGR weaned piglets through preventing AST and ALT from flowing into the circulatory system. These results indicated that IUGR induced liver injury and negatively influenced the growth performance of post-weaning piglets and curcumin supplementation could attenuate liver injury and improve the growth performance of IUGR weaned piglets.

Oxidative stress, which results from the imbalance of oxidation and antioxidation, can produce excessive free oxygen radicals, finally leading to lipid peroxidation and protein oxidation. Malondialdehyde is the primary product of lipid peroxidation. Hydrogen peroxide is well known for its deleterious effects on various cellular components [29]. In the present study, increased concentration of MDA indicated that the IUGR had a tendency to induce the imbalance of oxidation and antioxidation and lead to lipid peroxidation injury of newborn piglets. We also found that the IUGR significantly decreased the activities of antioxidant enzymes, increased the concentration of GSSG and the value of GSSG:GSH ratio in the liver of newborn piglets. Glutathione is one of the most important intracellular antioxidants that eliminates lipid peroxides and increases antioxidant capacity through the enzyme reactions catalyzed by GSH-Px [30]. During severe oxidative stress, GSH concentration decreases and converts into GSSG which is the disulfide form of GSH [31]. GR is also an important intracellular antioxidant enzyme that deoxidates GSSG to GSH under normal condition and protects cells against oxidative stress [32]. These results implied that IUGR decreased the metabolic efficiency of the GSH redox cycle in the liver of newborn piglets. It has been proven that the IUGR neonates are correlated with higher level of lipid peroxidation and lower levels of antioxidant enzyme activities and antioxidants [33]. Similar to the result of newborn piglets, IUGR markedly increased the concentrations of hepatic MDA and H_2_O_2_ in weaned piglets. In addition, we also found that the IUGR significantly decreased the activities of antioxidant enzymes in the serum and liver of weaned piglets. These findings were consistent with the results demonstrated by Zhang et al. [17], who found that IUGR resulted in oxidative stress and impaired antioxidant capacity in the serum and liver of fullly weaned piglets. Therefore, it is necessary to use nutritionally promising strategies in IUGR newborn piglets for the improvement of antioxidant status.

Curcumin is an antioxidant agent widely used to protect against oxidative stress and enhance antioxidant capacity in animal trials [9,34]. However, limited studies are available on the effect of curcumin on antioxidant capacity in IUGR piglets. The results of this study suggested that dietary supplementation of curcumin significantly decreased the concentrations of MDA and H_2_O_2_ and increased the activities of antioxidant enzymes in the serum or liver of IUGR weaned piglets. Similar findings were seen in the study conducted by Altintoprak [35], who found that dietary supplementation of curcumin was very helpful to increase antioxidant capacity and attenuate lipid peroxidation. The results of the present study suggested that curcumin could reduce the lipid peroxidation and increase antioxidant capacity in IUGR piglets. However, the mechanism related to the protective effect of curcumin on IUGR antioxidant capacity needs to be further investigated.

The activation of Nrf2/ARE (antioxidant response element) signaling plays a very important role in preventing cells from oxidative stress [36]. Under quiescent conditions, Nrf2 binds to actin-anchored protein Keap1. However, this quenching interaction will be released upon recognition of chemical signals imparted by oxidative stress. Then Nrf2 interacts with ARE and initiates antioxidant protection such as increasing activities of antioxidant enzyme, scavenging the oxygen free radicals, and regulating the expression of downstream genes. Hmox1, a sensitive redox protein, protects the body from various forms of stress when activated by Nrf2. In the present study, the mRNA expression levels for *Nfe2l2*, *Hmox1*, and downstream genes were decreased in the liver of IUGR newborn and weaned piglets, respectively. The protein expressions of Nrf2 and Hmox1 in the liver of IUGR newborn and weaned piglets were also obviously decreased in the present study. Our results implied that the impaired antioxidant capacity of IUGR newborn and weaned piglets may be resulted from the decreased mRNA and protein expressions of Nrf2 and Hmox1. Interestingly, dietary supplementation of curcumin was beneficial in upregulating the proteins expressions of Nrf2 and Hmox1, and mRNA expression levels for *Nfe2l2* and *Gpx1* in the liver of IUGR weaned piglets. Previous studies demonstrated that the curcumin supplementation could alleviate oxidative injury through Nrf2/Hmox1 pathway [37,38]. The current study, to the best of our knowledge, is the first to report that the supplementation of curcumin improved the antioxidant capacity of IUGR weaned piglets.

## 5. Conclusions

In conclusion, IUGR reduced body-weight gain and feed intake of weaned piglets and decreased activities of antioxidant enzymes in the liver of newborn and weaned piglets. Dietary curcumin supplementation could efficiently improve the body-weight gain and feed intake and increase activities of antioxidant enzymes and protein expressions of Nrf2 and Hmox1 in the liver of IUGR weaned piglets. Our findings may be helpful in finding a new nutritional therapeutic intervention for the early treatment of IUGR in animal production and human health.

## Figures and Tables

**Table 1:** Effect of curcumin on growth performance of intrauterine growth retardation weaned piglets (50 day).

Items	Experiment Groups				*p*-Value		
	NBW	NC	IUGR	IC	B	D	B × D
BWG (kg)	6.02 ± 0.04 ^a^	5.95 ± 0.08 ^ab^	4.44 ± 0.04 ^c^	5.31 ± 0.05 ^b^	<0.01	<0.01	<0.01
FI (kg)	7.23 ± 0.14 ^a^	7.45 ± 0.10 ^a^	5.13 ± 0.10 ^c^	6.17 ± 0.17 ^b^	<0.01	0.68	<0.01
G:F (kg/kg)	0.83 ± 0.02	0.80 ± 0.01	0.87 ± 0.01	0.86 ± 0.03	0.43	0.56	0.20

Values are means ± standard deviation; *n* = 8/group. Data were analyzed by using two-factor ANOVA and Tukey’s post hoc testing, where appropriate. Within a row, significant differences were marked with different letters when a significant interaction was observed (*p* < 0.05). NBW, normal birth weight; IUGR, intrauterine growth retardation; NC, NBW piglets with curcumin supplementation; IC, IUGR piglets with curcumin supplementation. Curcumin supplementation—piglets fed with diets supplemented with 400 mg/kg curcumin; B, birth weight; D, dietary curcumin supplementation; B × D was the interaction between the corresponding parameters. BWG, body-weight gain; FI, feed intake; G:F, feed conversion ratio, body-weight gain: feed intake.

**Table 2:** Effect of curcumin on the serum antioxidant enzymes activities of weaned piglets with intrauterine growth retardation (50 day).

Items	Experiment Groups				*p*-Value		
	NBW	NC	IUGR	IC	B	D	B × D
TAOC (U/mL)	1.94 ± 0.50 ^b^	2.08 ± 0.64 ^a^	1.03 ± 0.37 ^c^	1.89 ± 0.57 ^b^	0.01	0.01	0.06
CAT (U/mL)	4.68 ± 1.15 ^b^	6.88 ± 1.08 ^a^	1.77 ± 0.43 ^c^	3.59 ± 1.27 ^b^	<0.01	<0.01	0.61
GSH-Px (U/mL)	421.06 ± 63.75 ^a^	388.53 ± 38.06 ^ab^	355.29 ± 50.57 ^b^	376.87 ± 25.39 ^ab^	0.03	0.74	0.11
GR (U/L)	23.98 ± 2.73 ^bc^	25.79 ± 4.34 ^a^	21.47 ± 1.38 ^c^	30.14 ± 1.24 ^a^	0.35	<0.01	<0.01

Values are means ± standard deviation; *n* = 8/group. Data were analyzed by using two-factor ANOVA and Tukey’s post hoc testing, where appropriate. Within a row, significant differences are marked with different letters when a significant interaction was observed (*p* < 0.05). NBW, normal birth weight; IUGR, intrauterine growth retardation; NC, NBW piglets with curcumin supplementation; IC, IUGR piglets with curcumin supplementation. Curcumin supplementation—piglets fed with diets supplemented with 400 mg/kg curcumin; B, birth weight; D, dietary curcumin supplementation; B × D was the interaction between the corresponding parameters. CAT, catalase; TAOC, total antioxidant capacity; GSH-Px, glutathione peroxidase; GR, glutathione reductase.

**Table 3:** Effect of intrauterine growth retardation on the hepatic antioxidant enzyme activities of newborn piglets (0 day).

Items	NBW	IUGR	*p*-Value
TAOC (U/mg protein)	0.65 ± 0.10	0.48 ± 0.05	<0.01
CAT (U/mg protein)	12.66 ± 1.44	12.81 ± 1.43	0.83
GSH (µmol/g protein)	66.78 ± 17.84	80.78 ± 13.29	0.10
GSSG (µmol/g protein)	21.06 ± 2.69	17.92 ± 4.16	0.10
GSSG:GSH	0.22 ± 0.05	0.34 ± 0.13	0.04
GSH-Px (U/mg protein)	26.03 ± 0.70	22.43 ± 1.19	<0.01
GR (U/g protein)	15.13 ± 2.79	11.67 ± 3.47	0.03

Values are means ± standard deviation; *n* = 8/group. Data were analyzed using unpaired independent *t*-tests. Significant differences were observed while *p* < 0.05. NBW, normal birth weight; IUGR, intrauterine growth retardation. TAOC, total antioxidant capacity; CAT, catalase; GSH, glutathione; GSSG, oxidized glutathione; GSH-Px, glutathione peroxidase; GR, glutathione reductase.

**Table 4:** Effect of curcumin on the serum AST and ALT, and hepatic antioxidant enzymes activities of weaned piglets with intrauterine growth retardation (50 day).

Items	Experiment Groups				*p*-Value		
	NBW	NC	IUGR	IC	B	D	B × D
Serum							
AST (U/L)	21.65 ± 0.90 ^c^	18.69 ± 1.07 ^d^	25.93 ± 0.84 ^a^	23.27 ± 0.89 ^b^	0.02	0.01	0.07
ALT (U/L)	13.52 ± 1.53 ^c^	13.21 ± 1.43 ^c^	20.78 ± 1.67 ^a^	18.23 ± 1.97 ^b^	<0.01	0.07	0.03
Liver							
TAOC (U/mg protein)	1.00 ± 0.11 ^ab^	1.00 ± 0.06 ^ab^	0.88 ± 0.14 ^b^	1.21 ± 0.22 ^a^	0.37	<0.01	<0.01
CAT (U/mg protein)	7.92 ± 0.60 ^ab^	7.43 ± 0.55 ^b^	8.97 ± 0.79 ^a^	7.73 ± 0.63 ^b^	0.01	<0.01	0.12
GSH-Px (U/mg protein)	47.05 ± 6.73 ^b^	43.62 ± 7.36 ^b^	36.23 ± 2.04 ^c^	58.30 ± 5.17 ^a^	0.35	<0.01	<0.01
GR (U/g protein)	15.17 ± 1.27 ^b^	19.86 ± 3.57 ^a^	16.11 ± 1.22 ^b^	18.94 ± 3.36 ^a^	0.99	0.32	<0.01

Values are means ± standard deviation; *n* = 8/group. Data were analyzed by using two-factor ANOVA and Tukey’s post hoc testing, where appropriate. Within a row, significant differences are marked with different letters when a significant interaction was observed (*p* < 0.05). NBW, normal birth weight; IUGR, intrauterine growth retardation; NC, NBW piglets with curcumin supplementation; IC, IUGR piglets with curcumin supplementation. Curcumin supplementation—piglets fed with diets supplemented with 400 mg/kg curcumin; B, birth weight; D, dietary curcumin supplementation; B × D was the interaction between the corresponding parameters. AST, aspartate aminotransferase; ALT, alanine aminotransferase; CAT, catalase; TAOC, total antioxidant capacity; GSH-Px, glutathione peroxidase; GR, glutathione reductase.

**Table 5:** Effect of intrauterine growth retardation on the hepatic nuclear factor, erythroid 2-like 2 (*Nfe2l2*), heme oxygenase-1 (*Hmox1*), *Cat*, and glutathione peroxidase 1 (*Gpx1*) gene expressions of newborn piglets (0 day).

Items	NBW	IUGR	*p*-Value
*Nfe2l2*	1.00 ± 0.01	0.36 ± 0.03	<0.01
*Hmox1*	1.00 ± 0.03	0.76 ± 0.05	<0.01
*Cat*	1.00 ± 0.02	0.67 ± 0.02	<0.01
*Gpx1*	1.00 ± 0.04	0.69 ± 0.06	<0.01

Values are means ± standard deviation; *n* = 8/group. Data were analyzed using unpaired independent *t*-tests. Significant differences were observed while *p* < 0.05. *Nfe2l2*, nuclear factor, erythroid 2-like 2; *Hmox1*, heme oxygenase-1; *Cat*, catalase; *Gpx1*, glutathione peroxidase 1.

**Table 6:** Effect of curcumin on the hepatic nuclear factor, erythroid 2-like 2 (*Nfe2l2*), heme oxygenase-1 (*Hmox1*), *Cat*, and glutathione peroxidase 1 (*Gpx1*) gene expressions of weaned piglets with intrauterine growth retardation (50 day).

Items	Experiment Groups				*p*-Value		
	NBW	NC	IUGR	IC	B	D	B × D
*Nfe2l2*	1.00 ± 0.07 ^b^	1.25 ± 0.32 ^b^	0.57 ± 0.16 ^c^	2.04 ± 0.05 ^a^	0.01	<0.01	<0.01
*Hmox1*	1.00 ± 0.26 ^a^	1.05 ± 0.09 ^a^	0.54 ± 0.10 ^b^	0.59 ± 0.13 ^b^	<0.01	0.36	0.98
*Cat*	1.00 ± 0.26 ^b^	1.24 ± 0.11 ^a^	0.55 ± 0.10 ^c^	0.52 ± 0.05 ^c^	<0.01	0.06	0.02
*Gpx1*	1.00 ± 0.17 ^b^	1.15 ± 0.16 ^b^	0.60 ± 0.09 ^c^	1.76 ± 0.12 ^a^	0.05	<0.01	<0.01

Values are means ± standard deviation; *n* = 8/group. Data were analyzed by using two-factor ANOVA and Tukey’s post hoc testing, where appropriate. Within a row, significant differences were marked with different letters when a significant interaction was observed (*p* < 0.05). NBW, normal birth weight; IUGR, intrauterine growth retardation; NC, NBW piglets with curcumin supplementation; IC, IUGR piglets with curcumin supplementation. Curcumin supplementation—piglets fed with diets supplemented with 400 mg/kg curcumin; D, dietary curcumin supplementation; B × D was the interaction between the corresponding parameters. *Nfe2l2*, nuclear factor, erythroid 2-like 2; *Hmox1*, heme oxygenase-1; *Cat*, catalase; *Gpx1*, glutathione peroxidase 1.

## Supplementary Materials

The following are available online at https://www.mdpi.com/2072-6643/11/12/2978/s1, https://doi.org/10.5281/zenodo.3520037, Table S1: Composition of basal diets (as-fed basis), Table S2: Primer sequences used for quantitative real-time PCR assays.

## References

[1] Anand P., Kunnumakkara A.B., Newman R.A., Aggarwal B.B. (2007). Bioavailability of curcumin: Problems and promises. Mol. Pharm..

[2] Lao C.D., Ruffin M.T., Normolle D., Heath D.D., Murray S.I., Bailey J.M., Boggs M.E., Crowell J., Rock C.L., Brenner D.E. (2006). Dose escalation of a curcuminoid formulation. BMC Complement. Altern. Med..

[3] Toydemir T., Kanter M., Erboga M., Oguz S., Erenoglu C. (2015). Antioxidative, antiapoptotic, and proliferative effect of curcumin on liver regeneration after partial hepatectomy in rats. Toxicol. Ind. Health.

[4] Gan Z., Wei W., Li Y., Wu J., Zhao Y., Zhang L., Wang T., Zhong X. (2019). Curcumin and resveratrol regulate intestinal bacteria and alleviate intestinal inflammation in weaned piglets. Molecules.

[5] Wang M.E., Chen Y.C., Chen I.S., Hsieh S.C., Chen S.S., Chiu C.H. (2012). Curcumin protects against thioacetamide-induced hepatic fibrosis by attenuating the inflammatory response and inducing apoptosis of damaged hepatocytes. J. Nutr. Biochem..

[6] Ruby A.J., Kuttan G., Babu K.D., Rajasekharan K.N., Kuttan R. (1995). Anti-tumour and antioxidant activity of natural curcuminoids. Cancer Lett..

[7] Xun W., Shi L., Zhou H., Hou G., Cao T., Zhao C. (2015). Effects of curcumin on growth performance, jejunal mucosal membrane integrity, morphology and immune status in weaned piglets challenged with enterotoxigenic Escherichia coli. Int. Immunopharmacol..

[8] Wei S., Xu H., Xia D., Zhao R. (2010). Curcumin attenuates the effects of transport stress on serum cortisol concentration, hippocampal NO production, and BDNF expression in the pig. Domest. Anim. Endocrinol..

[9] Gao S., Duan X., Wang X., Dong D., Liu D., Li X., Sun G., Li B. (2013). Curcumin attenuates arsenic-induced hepatic injuries and oxidative stress in experimental mice through activation of Nrf2 pathway, promotion of arsenic methylation and urinary excretion. Food Chem. Toxicol..

[10] Hay W.W., Thureen P.J., Anderson M.S. (2001). Intrauterine growth restriction. NeoReviews.

[11] Wu G., Bazer F.W., Datta S., Gao H., Johnson G.A., Lassala A., Li P., Satterfield M.C., Spencer T.E. (2008). Intrauterine growth retardation in livestock: Implications, mechanisms and solutions. Arch. fur Tierz. Arch. Anim. Breed..

[12] Zohdi V., Lim K., Pearson J.T., Black M.J. (2015). Developmental programming of cardiovascular disease following intrauterine growth restriction: Findings utilising a rat model of maternal protein restriction. Nutrients.

[13] Amarilyo G., Oren A., Mimouni F.B., Ochshorn Y., Deutsch V., Mandel D. (2011). Increased cord serum inflammatory markers in small-for-gestational-age neonates. J. Perinatol..

[14] Mert I., Oruc A.S., Yuksel S., Cakar E.S., Buyukkagnici U., Karaer A., Danisman N. (2012). Role of oxidative stress in preeclampsia and intrauterine growth restriction. J. Obstet. Gynaecol. Res..

[15] Kamath U., Rao G., Kamath S.U., Rai L. (2006). Maternal and fetal indicators of oxidative stress during intrauterine growth retardation (IUGR). Indian J. Clin. Biochem..

[16] Liu J., Yao Y., Yu B., Mao X., Huang Z., Chen D. (2012). Effect of folic acid supplementation on hepatic antioxidant function and mitochondrial-related gene expression in weanling intrauterine growth retarded piglets. Livest. Sci..

[17] Hao Z., Yue L., Tian W. (2016). Antioxidant capacity and concentration of redox-active trace mineral in fully weaned intra-uterine growth retardation piglets. J. Anim. Sci. Biotechnol..

[18] NRC (2012). Nutrient Requirements of Swine.

[19] Dong L., Zhong X., Ahmad H., Li W., Wang Y., Zhang L., Wang T. (2014). Intrauterine growth restriction impairs small intestinal mucosal immunity in neonatal piglets. J. Histochem. Cytochem..

[20] Wang Y., Zhang L., Zhou G., Liao Z., Ahmad H., Liu W., Wang T. (2012). Dietary l-arginine supplementation improves the intestinal development through increasing mucosal Akt and mammalian target of rapamycin signals in intra-uterine growth retarded piglets. Br. J. Nutr..

[21] Zhang J., Xu L., Zhang L., Ying Z., Su W., Wang T. (2014). Curcumin Attenuates D-Galactosamine/Lipopolysaccharide-Induced Liver Injury and Mitochondrial Dysfunction in Mice. J. Nutr..

[22] He J., Dong L., Xu W., Bai K., Lu C., Wu Y., Huang Q., Zhang L., Wang T. (2015). Dietary tributyrin supplementation attenuates insulin resistance and abnormal lipid metabolism in suckling piglets with intrauterine growth retardation. PLoS ONE.

[23] Schmittgen T.D., Livak K.J. (2008). Analyzing real-time PCR data by the comparative C_T_ method. Nat. Protoc..

[24] Oztuna D., Elhan A.H., Tüccar E. (2006). Investigation of four different normality tests in terms of type 1 error rate and power under different distributions. Turk. J. Med. Sci..

[25] Biri A., Bozkurt N., Turp A., Kavutcu M., Himmetoglu O., Durak I. (2007). Role of oxidative stress in intrauterine growth restriction. Gynecol. Obstet. Investig..

[26] Xu W., Bai K., He J., Su W., Dong L., Zhang L., Wang T. (2015). Leucine improves growth performance of intrauterine growth retardation piglets by modifying gene and protein expression related to protein synthesis. Nutrition.

[27] Zhang J.F., Hu Z.P., Lu C.H., Yang M.X., Zhang L.L., Wang T. (2015). Dietary curcumin supplementation protects against heat-stress-impaired growth performance of broilers possibly through a mitochondrial pathway. J. Anim. Sci..

[28] Nyblom H., Berggren U., Balldin J., Olsson R. (2004). High AST/ALT ratio may indicate advanced alcoholic liver disease rather than heavy drinking. Alcohol Alcohol..

[29] Lee D.H., Lim B.S., Lee Y.K., Yang H.C. (2006). Effects of hydrogen peroxide (H_2_O_2_) on alkaline phosphatase activity and matrix mineralization of odontoblast and osteoblast cell lines. Cell Biol. Toxicol..

[30] Fang Y.Z., Yang S., Wu G. (2002). Free radicals, antioxidants, and nutrition. Nutrition.

[31] Jones D.P. (2002). Redox potential of GSH/GSSG couple: Assay and biological significance. Methods Enzymol..

[32] Mahmoud K.Z., Edens F.W. (2003). Influence of selenium sources on age-related and mild heat stress-related changes of blood and liver glutathione redox cycle in broiler chickens (*Gallus domesticus*). Comp. Biochem. Physiol. Part B Biochem. Mol. Biol..

[33] Hracsko Z., Orvos H., Novak Z., Pal A., Varga I.S. (2013). Evaluation of oxidative stress markers in neonates with intra-uterine growth retardation. Redox Rep. Commun. Free Radic. Res..

[34] Eldemerdash F.M., Yousef M.I., Radwan F.M. (2008). Ameliorating effect of curcumin on sodium arsenite-induced oxidative damage and lipid peroxidation in different rat organs. Food Chem. Toxicol..

[35] Altıntoprak N., Kar M., Acar M., Berkoz M., Muluk N.B., Cingi C. (2016). Antioxidant activities of curcumin in allergic rhinitis. Eur. Arch. Oto-Rhino-Laryngol..

[36] Kensler T.W., Wakabayashi N., Biswal S. (2007). Cell survival responses to environmental stresses via the Keap1-Nrf2-ARE pathway. Annu. Rev. Pharmacol. Toxicol..

[37] Joris M., Maartje D.V., Joris M., Anneke O., Stefaan D.S., Christa V.G. (2013). Maturation of digestive function is retarded and plasma antioxidant capacity lowered in fully weaned low birth weight piglets. Br. J. Nutr..

[38] Farombi E.O., Shrotriya S., Na H.K., Kim S.H., Surh Y.J. (2008). Curcumin attenuates dimethylnitrosamine-induced liver injury in rats through Nrf2-mediated induction of heme oxygenase-1. Food Chem. Toxicol..

