# Supplementary material for: Dietary Curcumin Supplementation Increases Antioxidant Capacity, Upregulates Nrf2 and Hmox1 Levels in the Liver of Piglet Model with Intrauterine Growth Retardation
Source: Nutrients. 2019 Dec 5;11(12):2978. doi: 10.3390/nu11122978 (PMC6950043; doi:10.3390/nu11122978)
Supplement: Supplementary file 1 [file nutrients-11-02978-s001.pdf]

nutrients

MDPI

Supplementary Tables

Table S1. Composition of basal diets (as-fed basis)

Ingredients	Ratio (%)	Nutrient composition	
Corn	57.70	Digestible energy (MJ/kg)	14.04
Soybean meal	12.50	Crude protein (%)	18.31
Expanded corn	8.00	Lysine (%)	1.31
Full-fat soybean	8.00	Methionine (%)	0.40
Soybean meal, fermented	4.00	Methionine + Cystine (%)	0.70
Whey powder	3.00	Threonine (%)	0.80
Fish meal (crude protein 67%)	3.00	Calcium (%)	0.85
Dicalcium phosphate	1.80	Total phosphorus (%)	0.72
Limestone	0.50		
L-lysine (78 %)	0.30		
L-threonine	0.10		
DL-methionine	0.08		
Wheat middling and reddog	0.02		
Premix[a]	1		
Total	100		

In the experiment 2, NBW and IUGR groups were fed with a basal diet, and the NC and IC groups were fed with a basal diet supplemented with 400 mg/kg curcumin.

[a]The premix supplied the following per kg complete diet: vitamin A, 12000 IU; vitamin D_3, 3000 IU; α-tocopherol, 50 mg; vitamin K_3, 4 mg; vitamin B_1, 4 mg; vitamin B_2, 10 mg; vitamin B_6, 7 mg; vitamin B_{12}, 0.05 mg; niacin, 30 mg; pantothenic acid, 15 mg; folic acid, 0.3 mg; biotin, 0.08 mg; choline chloride, 500 mg; Fe ($FeSO4 \cdot H_2O$), 110 mg; Cu ($CuSO_4 \cdot 5H_2O$), 7 mg; Zn (ZnO), 110 mg; I (KIO_3), 0.3 mg; Mn ($MnSO4 \cdot H_2O$), 5 mg; Se (Na_2SeO_3), 0.3 mg.

Table S2. Primer sequences used for quantitative real-time PCR assays

Gene	Accession no.	Primer, 5′-3′
Nfe2l2	NM_006164.4	CTCCGGGTGTGTTTGTTCCA
		GTTGTTTGCGAAGGTCGCTG
Hmox1	NM_001004027.1	CAACCCTGTGAATGCAACCG
		CACATGCCCAACAAGGAAGC
Cat	NM_214301.2	TGTGAACTGTCCCTTCCGTG
		CGTCTGTTCGGGAGCACTAA
Gpx1	NM_214201.1	TGGGGAGATCCTGAATTGCC
		CGAAGAGCATGAAGTTGGGC
Actb	XM_003124280.4	CAGTCGGTTGGATGGAGCAT
		AGGCAGGGACTTCCTGTAAC

Nfe2l2, nuclear factor, erythroid 2-like 2; Hmox1, Haeme oxygenase-1; Cat, catalase; Gpx1, glutathione peroxidase 1; Actb, β-actin.

© 2019 by the authors. Licensee MDPI, Basel, Switzerland. This article is an open access article distributed under the terms and conditions of the Creative Commons Attribution (CC BY) license (http://creativecommons.org/licenses/by/4.0/).
